# The *Botrytis cinerea* Xylanase BcXyl1 Modulates Plant Immunity

**DOI:** 10.3389/fmicb.2018.02535

**Published:** 2018-10-23

**Authors:** Yuankun Yang, Xiufen Yang, Yijie Dong, Dewen Qiu

**Affiliations:** The State Key Laboratory for Biology of Plant Diseases and Insect Pests, Institute of Plant Protection, Chinese Academy of Agricultural Sciences, Beijing, China

**Keywords:** *Botrytis cinerea*, xylanase, BcXyl1, plant immunity, virulence

## Abstract

*Botrytis cinerea* is one of the most notorious pathogenic species that causes serious plant diseases and substantial losses in agriculture throughout the world. We identified BcXyl1 from *B. cinerea* that exhibited xylanase activity. Expression of the *BcXyl1* gene was strongly induced in *B. cinerea* infecting *Nicotiana benthamiana* and tomato plants, and *BcXyl1* deletion strains severely compromised the virulence of *B. cinerea*. BcXyl1 induced strong cell death in several plants, and cell death activity of BcXyl1 was independent of its xylanase activity. Purified BcXyl1 triggered typically PAMP-triggered immunity (PTI) responses and conferred resistance to *B. cinerea* and TMV in tobacco and tomato plants. A 26-amino acid peptide of BcXyl1 was sufficient for elicitor function. Furthermore, the BcXyl1 death-inducing signal was mediated by the plant LRR receptor-like kinases (RLKs) BAK1 and SOBIR1. Our data suggested that BcXyl1 contributed to *B. cinerea* virulence and induced plant defense responses.

## Introductions

*Botrytis cinerea* is a necrotrophic pathogen, causing widespread plant diseases and enormous economic losses in a large number of important crops throughout the world (Prins et al., 2000). *B. cinerea* can infect various organs in plants, including leaves, bulb, flowers, fruits, and root tubers. The infection process of *B. cinerea* mainly includes two typical stages: local lesions at an early stage and a late stage of fast-spreading lesions.

The plant cell wall is a natural barrier, which provides mechanical strength and rigidity to prevent pathogen infection. To establish successful colonization, *B. cinerea*, like other fungal pathogen, secretes a large number of cell wall-degrading enzymes (CWDEs) to degrade the plant defensive barriers during the infection process, thereby to permit pathogens to invade plant tissue and supply pathogens with nutrients (Cantarel et al., 2009; Kubicek et al., 2014). These CWDEs, including pectinases, cellulases, cutinases, and xylanases, are generally regarded as important virulence factors through the maceration of host tissues and the degradation of host macromolecules (Prins et al., 2000). The effects of targeted deletion of some genes encoding CWDEs support their direct involvement in the infection process. For example, deletion of the pectate lyase gene *CcpelA* and the pectate lyase gene *PelB* in *Colletotrichum coccodes*, resulted in a substantial loss of virulence on green tomato fruit and reduced virulence on avocado, respectively (Yakoby et al., 2001; Ben-Daniel et al., 2011). Targeted deletion of VdCUT11, a cutinase in *V. dahliae*, significantly compromised virulence on cotton plants (Gui Y. et al., 2017). However, the specific roles of the majority of CWDEs in pathogen virulence remain largely unknown, especially in *B. cinerea*.

To ward off microorganisms infection, plants have evolved elaborate systems to provide better immunity against pathogens (Zipfel, 2008). Recognition of conserved pathogen-associated molecular patterns (PAMPs) *via* pattern recognition receptors (PRRs) located on the cell surface constitutes the first layer of plant innate immunity and is termed as PAMP-triggered immunity (PTI). Intracellular responses associated with PTI include Ca^2+^ influx, the burst of reactive oxygen species (ROS), the accumulation of defense hormone, the expression of defense-related genes and callose deposition (Boller and Felix, 2009; Couto and Zipfel, 2016). In turn, during the coevolution of hosts and microbes, pathogens also employ numerous effectors to interfere with PTI and establish successful infection, which is regarded as effector-triggered susceptibility (ETS) (Chisholm et al., 2006; Jones and Dangl, 2006; Saijo et al., 2017). As a countermeasure, some plants recruit R proteins to recognize these effectors directly or indirectly termed effector-triggered immunity (ETI) (Houterman et al., 2008; Stergiopoulos and de Wit, 2009). Generally, ETI is often accompanied with stronger immune responses, such as hypersensitive response (HR).

Apart from the role of virulence factor, some CWDEs also function as PAMPs to activate the plant immune responses independent of their enzymatic activity. For instance, VdEG1, VdEG3 and VdVUT11 from *Verticillium dahliae*, XEG1 from *Phytophthora sojae* and BcXYG1, a secreted xyloglucanase from *B. cinerea* contributed to virulence and triggered plant immunity as PAMPs simultaneously (Ma et al., 2015; Gui Y. et al., 2017; Gui Y.-J. et al., 2017; Zhu et al., 2017).

Plants recognizes characteristic microbial molecules classically known as PAMPs by employing a multitier surveillance system, including PRRs (Couto and Zipfel, 2016). Plant PRRs include RLKs and receptor-like proteins (RLPs) (Boutrot and Zipfel, 2017). Currently, a handful of PRRs have been identified as receptors to participate in the recognition of PAMPs. The brassinosteroid insensitive 1 (BRI1)-associated receptor kinase 1 (BAK1) and the LRR receptor-like kinase (LRR-RLK) SUPPRESSOR OF BIR1-1 (SOBIR1) are involved in multiple PRR pathways and signal activation (Liebrand et al., 2014). For example, BcSpl1, XEG1, and VdCUT11 could trigger cell death in the plants, and the resulting immunity signal was mediated by the plant LRR RLKs BAK1 and SOBIR1 (Frías et al., 2011; Ma et al., 2015; Gui Y. et al., 2017).

Xylan is the major component of hemicellulose of the plant cell wall (Collins et al., 2005). Due to the complexity, the degradation of xylan requires several hydrolytic enzymes, of which xylanase is a crucial component for hydrolyzing the 1,4-β-d-xylosidic linkages in xylan. Xylanase has received more attention because of the special role in fungi pathogenicity. For example, a mutation in the *xynB* endoxylanase gene from *Xanthomonas oryzae* pv. *oryzae* resulted in attenuated virulence in rice (Pandey and Sonti, 2010). Moreover, the deletion of xylanases *Xyn11A* gene had a marked effect on the ability of *B. cinerea* to infect tomato leaves and grape (Brito et al., 2006). In addition to their roles in virulence, xylanases are regarded as elicitors to induce defense responses in plants. For example, ethylene-inducing xylanase (EIX) is a potent elicitor in tobacco and tomato. However, the function of the majority of xylanases in *B. cinerea* remains mostly undiscovered. Here, we reported on the identification and characterization of BcXyl1, a xylanase from *B. cinerea*. BcXyl1 contributes to *B. cinerea* virulence and triggers PTI responses in plants. Furthermore, a small peptide of BcXyl1 is sufficient for elicitor function. We found that the cell death signal is mediated by BAK1 and SOBIR1, and the xylanases activity is not necessary for the induction of necrosis.

## Materials and Methods

### Fungal Cultures, Plants Grown

*Botrytis cinerea* B05.10 was used as wild-type strain and control strain in this study. All *B. cinerea* strains, including two independent *BcXyl1* knockout mutants and two complementary transformants, were routinely maintained in 15% glycerol at -80°C and grown on PDA at 22°C, respectively. *Agrobacterium tumefaciens* AGL-1 were grown on LB (Kan and Rif) medium at 28°C. To obtain conidia, *B. cinerea* grown on tomato-PDA plates (39 g of potato dextrose agar plus 250 g of homogenized tomato fruits per liter) as explained previously (Benito et al., 1998). *N. benthamiana* and tomato (*Solanum lycopersicum*) plants were grown at 27 °C in a greenhouse with a day/night period of 14/10 h and 60% relative humidity (RH).

### Expression and Purification of Recombinant Protein

The open reading frame of *BcXyl1* (amplified with primers BcXyl1 F/BcXyl1 R; Supplementary Table S2) and C^130-155^ were amplified by PCR from cDNA of the wild-type strain B05.10 and the fragment fused with a myc tag and a 6xHis tag at the C terminus was cloned into the pPICZαA vector at the *Bam*HI and *Eco*RI sites. The recombinant plasmid pPICZαA-BcXyl1 and pPICZαA-C^130-155^ were linearized with *Pme*I and transformed into *Pichia pastoris* KM71H for expression. The transformed yeasts were grown and induced in BMGY (buffered glycerol complex medium) and BMMY (buffered methanol complex medium), respectively (Easy Select Pichia expression kit; Invitrogen). Then, the supernatant was collected (3000 *g* for 10 min at 4°C) and purified using nickel affinity chromatography. The purified C^130-155^, BcXyl1^rec^, or BcXyl1 were kept in protein buffer (20 mM Tris, pH 8.0) and further detected *via* SDS-PAGE and Western blotting. The concentration of the purified protein was measured using Easy II Protein Quantitative Kit (BCA) and the protein was then stored at -80°C.

### Truncated Mutant Construction and Agroinfiltration Assay

To transiently express truncated mutants of the BcXyl1 protein in leaves, DNA sequences encoding different fragments (BcXyl1, N^80^, N^130^, N^155^ C^80^, C^130^, C^155^, and C^130-155^) were amplified by PCR from cDNA of the wild-type strain B05.10 and inserted into pYBA1132 vector at the *Xba*I and *Bam*HI sites and then transformed into the *A. tumefaciens* strain GV3101. Agroinfiltration assays were performed on *N. benthamiana* plants. *Agrobacterium*-mediated transient expression was performed as described (Ma et al., 2015). Leaves were scored and photographed 6 days after initial inoculation. Each assay was performed on six leaves from three individual plants, and repeated at least three times.

### Site-Directed Mutagenesis

To determine the relationship between the enzymatic activity and cell death-inducing activity of BcXyl1, we constructed BcXyl1^rec^ mutant, which abolished the enzymatic activity. According to multiple sequence alignment, two potentially highly conserved catalytic residues (E104 and E157) were the critical catalytic sites of BcXyl1. Next, two glutamic acid residues were substituted by Gln using the Quick Change^TM^ Site-Directed Mutagenesis Kit (Stratagene, United States). BcXyl1^rec^ was expressed in *P. pastoris* and carried out the enzyme assay.

### Xylanase Assay

The xylanase activity was assayed *via* the method as described previously (Biely et al., 1988). The purified BcXyl1^rec^ or BcXyl1 (500 ng) and substrate (1% beechwood xylan) were co-incubated in citrate phosphate McIlvaine buffer, pH 5, at 35°C for 10 min (total volume: 125 μl). All samples were incubated at 100°C for 10 min to end the assays reactions. The amount of reducing sugars released from xylan was quantified using a standard calibration curve obtained with the dinitrosalicylic acid procedure. The experiment was replicated three times.

### Immunoblot Analysis

To confirm whether BcXyl1 was secreted into the apoplast and the relationship between enzymatic activity of BcXyl1 and cell death-inducing activity, transient expression in *N. benthamiana* was performed. Three sequences (BcXyl1, BcXyl1^-ΔSP^, and BcXyl1^rec^) were cloned into the pYBA1132 vector which contained a C-terminal GFP tag at the *Xba*I and *Bam*HI sites, and then transformed into the *A. tumefaciens* strain GV3101. All primers are listed in Supplementary Table S2. Plant total protein extractions and immunoblots were assessed as previously described (Yu et al., 2012). All proteins were analyzed by immunoblots using anti-GFP-tag primary monoclonal antibody. The blots were visualized using the Odyssey^®^ LI-COR Imaging System. Rubisco was used to confirm the equal protein loading.

### Protein Infiltration Assays and Induction of PTI by BcXyl1

To test the induction of cell death or PTI responses, BcXyl1 and C^130-155^ were dissolved in PBS and infiltrated into the leaves of *N. benthamiana* and tomato plants using a syringe. Plants were grown in a greenhouse with a day/night period of 14/10 h. The cell-death response was investigated after 48 h treated with BcXyl1, C^130-155^, or PEVC (*P. pastoris* culture supernatant from an empty vector control strain, purified in the same way as BcXyl1). To further investigate cell death, trypan blue staining was performed by boiling leaf tissues in a mixture of phenol, lactic acid, glycerol, and distilled water containing 1 mg/ml trypan blue (1:1:1:1) for 1 min. The samples were then soaked in 2.5 mg/ml chloral hydrate overnight. The accumulation of ROS in plant leaves was stained by 3′3-diaminobenzidine (DAB) and Nitroblue Tetrazolium (NBT) solution as described previously (Bindschedler et al., 2006). To visualize callose deposition, 4-week-old *N. benthamiana* leaves were infiltrated with 1 μM recombinant proteins and stained with aniline blue at 24 h post-treatment, as described previously (Chen et al., 2012). To assay electrolyte leakage, the *N. benthamiana* leaves treated with proteins were harvested at different time points and submerged in sterile water at 4°C. Ion conductivity was measured using a conductivity meter. To test whether BcXyl1 could confer plants disease resistance, the purified BcXyl1, C^130-155^, or PEVC was individually syringe-infiltrated into 4-week-old *N. benthamiana* and tomato leaves. Five microliters of 2 × 10^6^ conidia/ml *B. cinerea* and TMV-GFP were placed on the systemic leaves, respectively. The inoculated plants were placed in a greenhouse at 25°C with a day/night period of 14/10 h. Lesion diameter of *B. cinerea* and the number of TMV-GFP lesions on *N. benthamiana* leaves were evaluated at 2 and 4 days post-inoculation, respectively. All experiments were performed three times.

### Pathogenicity Assays

To test whether BcXyl1 functioned as a virulence factor of *B. cinerea*, the wild-type strain and derived mutants, including the BcXyl1 deletion (Δ*BcXyl1*-1 and Δ*BcXyl1*-2) and complementary mutants (Δ*BcXyl1*-1-C and Δ*BcXyl1*-2-C) were used in this study. Four-week-old *N. benthamiana* leaves were inoculated with 5 μL of 2 × 10^6^ conidia/ml *B. cinerea*. The inoculated plants were placed in a greenhouse with a day/night period of 14/10 h. The lesion development of *B. cinerea* on the *N. benthamiana* leaves was evaluated at 2 days post-inoculation by determining the average lesion diameter. Tomato, grape, and apple fruits (commercially obtained) were washed under running tap water and surface sterilized by immersion for 5 min in ethanol. After air drying, fruits were inoculated with 5 μL of 2 × 10^6^ conidia/ml *B. cinerea*. Fruits were incubated at 25°C under conditions of high humidity on water-soaked filter paper in closed containers. The lesion development of *B. cinerea* on the fruits was evaluated at 3 days post-inoculation by determining the average lesion diameter. All the experiments were performed three times.

### VIGS in *N. benthamiana*

To determine whether BAK1 or SOBIR1 participate in induction of cell death by BcXyl1, VIGS was performed. *NbBAK1* or *NbSOBIR1* gene was silenced using VIGS, as described previously (Kettles et al., 2016). *A. tumefaciens* strain harboring constructs (pTRV1, pTR::*BAK1* or pTRV1, pTRV2::*SOBIR1*) were infiltrated into the *N. benthamiana* leaves. pTRV2::*GFP* was used as the control and the expression levels of *BAK1* and *SOBIR1* were determined by qRT-PCR. Agroinfiltration assays were performed on *N. benthamiana* plants using Bcl-2-associated X protein (BAX) as positive controls. Phenotypes were photographed 6 days after infiltration. All the experiments were performed three times.

### RNA Extraction and qRT-PCR

To measure the expression of *BcXyl1* during infection, 4-week-old *N. benthamiana* or 4-week-old tomato plants were inoculated with *B. cinerea* 2 × 10^6^ conidia/ml. We selected 10 indicated time points during different stages of post-inoculation to determine expression patterns of *BcXyl1* by qPCR. All samples were stored at -80°C. Total RNA of *B. cinerea* was extracted with the E.Z.N.A.^®^ Total RNA Kit I according to the manufacturer’s instructions and stored at -80°C. For the measurement of defense-related genes expression, leaves of 4-week-old *N. benthamiana* plants were treated with 1 μM purified BcXyl1, C^130-155^, or PEVC. The leaves were obtained at the indicated time points, immediately frozen in liquid nitrogen, and stored at -80°C. The EasyPure Plant RNA Kit (TransGen Biotech) was used to extract total RNA. After isolation of total RNA, qPCR was performed using a TransStart Green qPCR SuperMix UDG according to the manufacturer’s instructions (TransGen Biotech). qRT-PCR was performed under the following conditions: an initial 95°C denaturation step for 10 min followed by 40 cycles of 95°C for 15 s and 60°C for 1 min. *N. benthamiana* EF-1a (P43643.1) and *B. cinerea Bcgpdh* gene (BC1G_05277) were used as endogenous plant controls and used to quantify fungal colonization, respectively. qPCR assays were repeated at least twice, each repetition with three independent replicates (Livak and Schmittgen, 2001). All primers are listed in Supplementary Table S2. The relative transcript levels among various samples were determined using the 2^-ΔΔCT^ method with three independent determinations (Livak and Schmittgen, 2001).

### Generation of *BcXyl1* Deletion and Complementary Mutants

*BcXyl1* gene and 500 bp flanking sequences of the target gene were amplified from *B. cinerea* B05.10 wild-type strain genomic DNA. Two flanking sequences of the target gene and hygromycin resistance cassette were constructed into a fusion fragment using a nested PCR reaction, which is subsequently introduced into the binary vector pGKO2 gateway. To generated complementary transformants, the donor vector pCT-HN containing *BcXyl1* gene was integrated into the mutant transformants using a previously described *Agrobacterium*-mediated transformation method (Liu et al., 2013). All mutants were identified using PCR with the corresponding primers. All primers are listed in Supplementary Table S2.

### Statistical Analysis

All the experiments and data presented here were performed at least three repeats. The data are presented as the means and standard deviations. Statistical Analysis System (SAS) software was used to perform the statistical analysis *via* Student’s *t*-test.

## Results

### Amino Acid Sequence Analysis of VdCP1 BcXyl1

BcXyl1 was identified by searching the *B. cinerea* genome sequence. The open reading frame of BcXyl1 (GenBank: ATZ53308.1) is 987 bp encoding a 329 aa protein with a predicted N-terminal signal peptide (1–20 aa), and no transmembrane helices of BcXyl1 were found, suggesting that it may be secreted into extracellular space. The bioinformatics analysis suggested that BcXyl1 belongs to SGNH hydrolase subfamily and has a highly strong similarity to fungal endo-β-1,4-xylanases.

### BcXyl1 Contributes to *B. cinerea* Virulence

**FIGURE 1:**
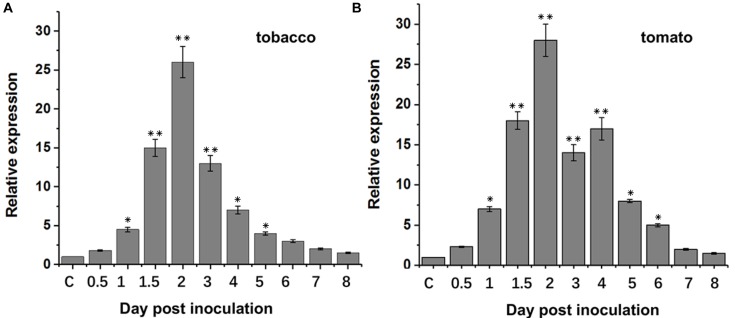
*BcXyl1* expression analysis during infection of tobacco and tomato plants. Tobacco and tomato leaves were inoculated with *B. cinerea* spores, and the expression of *BcXyl1* was detected by qPCR. The control (C) was mixed with non-inoculated conidia tobacco or tomato leaves. *B. cinerea Bcgpdh* gene (BC1G_05277) was used as an endogenous control. Error bars represent standard deviation of three independent replicates. Asterisks indicate significant differences with based on Student’s *t*-test (^∗^*p* < 0.05 and ^∗∗^*p* < 0.01).

Previous studies showed that xylanases in pathogenic microorganisms were implicated in the pathogenicity. In order to assess the role of BcXyl1 to *B. cinerea* virulence, we first analyzed the expression patterns of *BcXyl1* during different stages of post-inoculation. qRT-PCR results suggested that when the spore suspension of *B. cinerea* was inoculated onto leaves of *N. benthamiana* and tomato, transcript level of *BcXyl1* increased rapidly and reached a maximum of about 26-fold to 28-fold at 2 days post-inoculation, and then rapidly declined and maintained a level that was slightly higher than the initial level during later stages (Figure 1).

**FIGURE 2:**
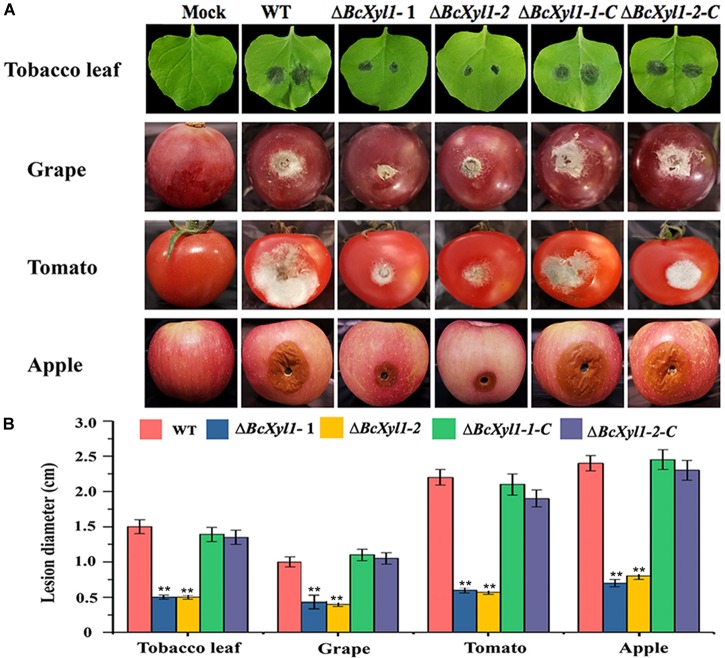
Virulence analysis of *BcXyl1* mutants on plant organs. **(A)** Disease symptoms on wounded tobacco leaves, grape fruits, tomato fruits, and apple fruits after 72 h inoculation. **(B)** Diameter of disease lesion on leaves and fruits was determined. Error bars represent standard deviation of three independent replicates. Student’s *t*-test was performed to determine the significant differences between mutants and WT stain. Asterisks “^∗∗^” indicate statistically significant differences at a *p*-value <0.01.

To more directly explore the biological roles of BcXyl1 during infection, we constructed two *BcXyl1* deletion mutants in *B. cinerea* (Δ*BcXyl1*-1 and Δ*BcXyl1*-2) and two rescued strains (Δ*BcXyl1*-1-C and Δ*BcXyl1*-2-C), and the ability of the resulting mutants to infect various plant organisms was evaluated. All mutants showed no significant differences with the wild-type stain in growth rate and colony morphology on PDA plates (Supplementary Figure S2). *N. benthamiana* leaves were inoculated with spore suspension of the wild type and mutants, and lesion size was measured 48 h after inoculation. Interestingly, the deletion of *BcXyl1* displayed significantly reduced virulence and produced much smaller lesions on leaves of *N. benthamiana* than the WT strain at 48 hpi (Figures 2A,B). The rescued strains recovered the high virulence phenotypes. And two *BcXyl1* deletion mutants displayed much weaker disease symptoms and lesion diameter than the wild-type strain and the complement strains (Δ*BcXyl1*-1-C and Δ*BcXyl1*-2-C) on grape, tomato, and apple fruits 72 h post-inoculation (Figures 2A,B). These results indicated that BcXyl1 functioned as a virulence factor that contributes to *B. cinerea* virulence on host plants.

### BcXyl1 Is a Secreted Protein to Induce Cell Death in Several Plant Species

**FIGURE 3:**
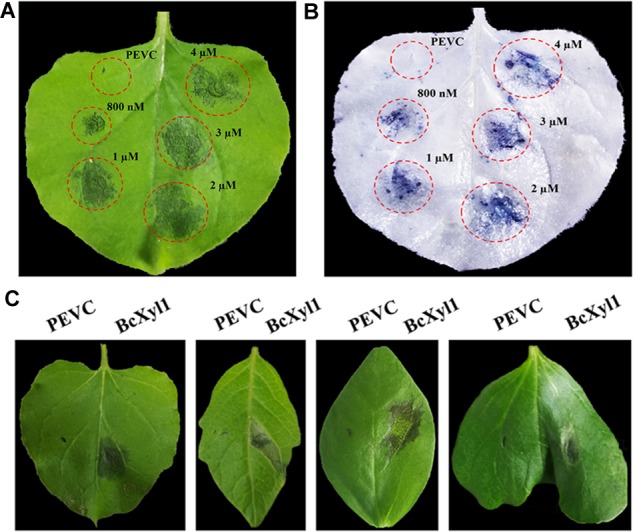
BcXyl1 induces cell death in several plants. **(A,B)** *N. benthamiana* leaves were infiltrated with purified BcXyl1 protein (800 nM to 2 μM), and PEVC (*P. pastoris* culture supernatant from an empty vector control strain, purified in the same way as BcXyl1). Two days post-infiltration, the leaves were photographed and stained with trypan blue. **(C)** The leaves of tomato, soybean, and cotton were infiltrated with purified BcXyl1 (1 μM) and PEVC (1 μM). Two days post-infiltration, different plants leaves were photographed. All the experiments were replicated three times.

To further confirm whether BcXyl1 could induce cell death in *N. benthamiana*, we expressed BcXyl1 in the yeast *P. pastoris* using the pPICZαA vector (pPICZαA: *BcXyl1*). Moreover, the recombinant protein BcXyl1, with a size of 35 kDa, was infiltrated into the mesophyll of *N. benthamiana* leaves with different concentrations (Supplementary Figure S3). The area of necrosis occurred and increased with increasing concentrations of BcXyl1 from 800 nM to 2 μM after infiltration 3 days, whereas no cell death activity was detected in the leaves treated with PEVC (*P. pastoris* culture supernatant from an empty vector control strain, purified in the same way as BcXyl1) (Figures 3A,B).

To examined the cell death-inducing activity of BcXyl1 in plants other than *N. benthamiana*, we infiltrated BcXyl1 (1 μM) into the leaves of several plants, including tomato, soybean, and cotton. BcXyl1 could induce significant cell death in these plants while PEVC did not (Figure 3C). So, BcXyl1 has ability to induce cell death in several plant species.

BcXyl1 has a signal peptide with 20 amino acids and no transmembrane helices, implying that BcXyl1 might be a secreted protein. In order to check if, as previously hypothesized, BcXyl1 was secreted into the apoplast to induce cell death response, we transiently expressed the full length BcXyl1 and BcXyl1^-ΔSP^ (lacking the signal peptide) in *N. benthamiana* by agroinfiltration. The results showed that BcXyl1 containing signal peptide induced cell death in *N. benthamiana*, whereas BcXyl1^-ΔSP^ lacking signal peptide abolished the ability to trigger cell death at 5 days after agroinfiltration (Supplementary Figure S4A). The protein expression level of BcXyl1 and BcXyl1^-ΔSP^ in *N. benthamiana* were detected by immunoblot (Supplementary Figure S4B). So, all results showed BcXyl1 was delivered into the apoplast to induce cell death in several plant species.

### The Cell Death-Inducing Activity Is Independent of the Xylanase Activity of BcXyl1

**FIGURE 4:**
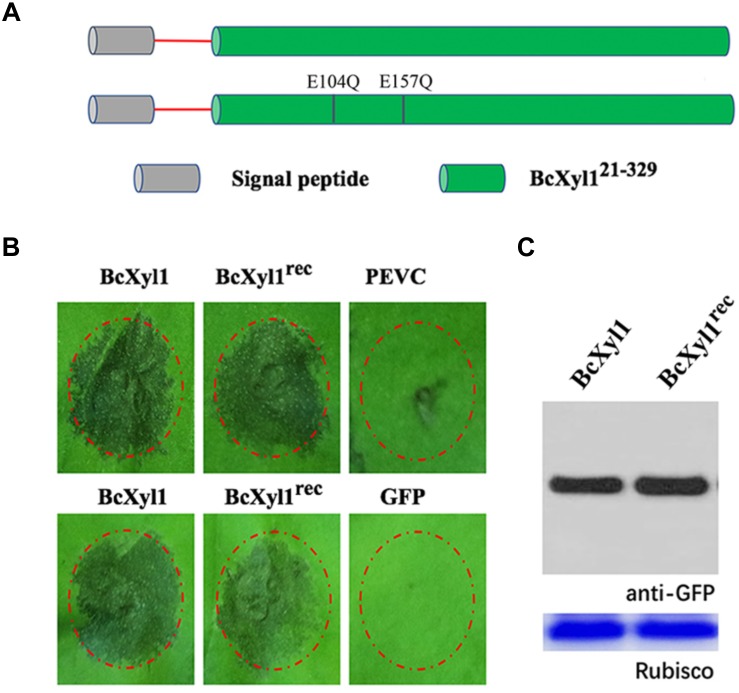
Cell death activity of BcXyl1 is independent of its xylanase activity in *N. benthamiana*. **(A)** Schematic presentation of the examined constructs. BcXyl1 (the native protein) and BcXyl1^rec^ (replaced E104 and E157 with Gln). **(B)** Upper pictures: treatment of tomato leaves with 1 μM purified BcXyl1 or BcXyl1^rec^. Lower pictures: analysis of cell death produced by *A. tumefaciens* strains transiently expressing BcXyl1 or BcXyl1^rec^. **(C)** Immunoblot analysis of proteins from *N. benthamiana* leaves transiently expressing the examined proteins using a pYBA1132 vector. All the experiments were replicated three times.

Previous reports showed that xylanases from fungi had ability to degrade xylan (Brutus et al., 2005). Interestingly, purified BcXyl1 had a xylanase activity using low viscosity xylane (LVX) as substrate (Supplementary Table S1). The sequence alignment results showed that BcXyl1 included two potentially highly conserved catalytic residues (E104 and E157), which are essential for the xylanase activity (Supplementary Figure S1). In addition, the enzymatic activity of CWDEs was required for cell death activity, and in a few cases, the cell death-inducing activity was found to be independent of the enzymatic activity. To determine the relationship between the enzymatic activity and cell death-inducing activity of BcXyl1, we generated a site-directed mutant (BcXyl1^rec^) that two glutamic acid residues were substituted by Gln using site-directed mutagenesis and expressed the mutant protein in *P. pastoris* (Figure 4A). Enzymatic assays with purified BcXyl1^rec^ showed the xylan-degrading xylanase activity was abolished (Supplementary Table S1). Surprisingly, although BcXyl1^rec^ absent the ability of xylanase activity, retained the same cell death-inducing activity as the wild-type (BcXyl1) (Figure 4B). Further, *A. tumefaciens* infiltration assays showed that BcXyl1^rec^ and BcXyl1 induced similar visible cell death symptoms in *N. benthamiana* leaves 4 days post-inoculation (Figure 4B). Western blot assays showed that the accumulation of BcXyl1 and BcXyl1^rec^ was similar (Figure 4C). These results confirmed that BcXyl1 did not need to xylanase activity to induce cell death in *N. benthamiana*.

### BcXyl1 Triggers the PTI Responses

**FIGURE 5:**
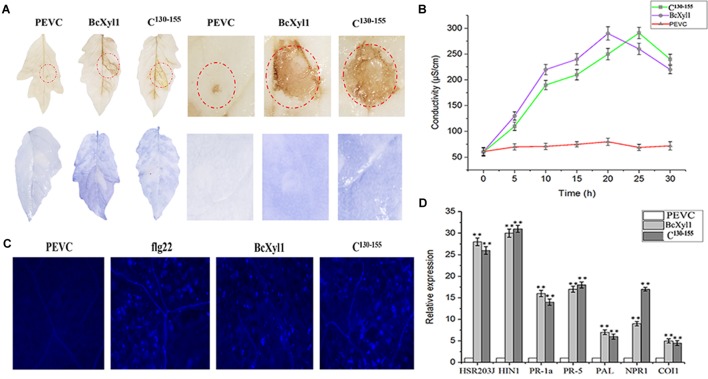
BcXyl1 induces PTI responses in plants. *N. benthamiana* or tomato leaves were infiltrated with 1 μM purified BcXyl1, C^130-155^, or PEVC. **(A)** ROS accumulation was detected in tobacco and tomato leaves 12 h after infiltration. The treated leaves were stained with DAB and NBT. All the experiments were replicated three times. **(B)** The conductivity was measured at the indicated time points. Error bars represent standard errors. All the experiments were replicated three times. **(D)** The expression of defense response genes was measured in *N. benthamiana* leaves by qPCR. Error bars represent standard deviation of three independent replicates. Student’s *t*-test was performed to determine the significant differences between BcXyl1, C^130-155^, and PEVC. Asterisks “^∗∗^” indicate statistically significant differences at a *p*-value <0.01. **(C)** Callose deposition in *N. benthamiana* leaves were detected 2 days after infiltration; the treated leaves were stained with aniline blue. All the experiments were replicated three times.

Some cell death-inducing proteins are recognized by plant immune system and activate host PTI responses, bring a series of typical characteristics such as accumulation of ROS, leakage of ion electrolytes, expression of defense genes, and callose deposition (Frías et al., 2012; Zhang et al., 2014, 2015). To examine whether BcXyl1 could induce typical PTI responses, the leaves of *N. benthamiana* and tomato plants were infiltrated with 1 μM BcXyl1. The ability of BcXyl1 to induce the accumulation of ROS in the infiltrated leaves was studied. The hydrogen peroxide (H_2_O_2_) and superoxide anion (O_2-_) production levels were assayed using DAB and NBT, respectively. A clear brown and blue precipitate was observed in leaves treated with BcXyl1, whereas the leaves treated with PEVC showed opposite patterns of DAB and NBT signal (Figure 5A). Meanwhile, BcXyl1 also induced electrolyte leakage and displayed an increase in conductivity, while PEVC exhibited barely change at the same concentration (Figure 5B). BcXyl1 was shown to cause significantly upregulation of seven genes associated with PTI and defense response in *N. benthamiana* leaves 12 h after treatment with BcXyl1; these genes included *PR-1a* and *PR-5*, which are involved in the SA-dependent defense pathway, *PAL* (phenylalanine ammonia lyase), *NPR1* (the non-expressor of pathogenesis related 1), *HSR203J* and *HIN1*, which are two HR marker genes in *tobacco*, and *COI1* (CORONATINE INSENSITIVE 1), which is JA responsive (Figure 5C). We finally examined callose deposition in leaves treated with BcXyl1, PEVC, or flg22. Furthermore, *N. benthamiana* leaves infiltrated with BcXyl1 or flg22 exhibited strong callose deposition compared with those infiltrated with PEVC, which exhibited undetectable levels of callose deposition (Figure 5D). These data indicated that BcXyl1 could induce typical PTI responses.

### BcXyl1 Confers Plants Disease Resistance

**FIGURE 6:**
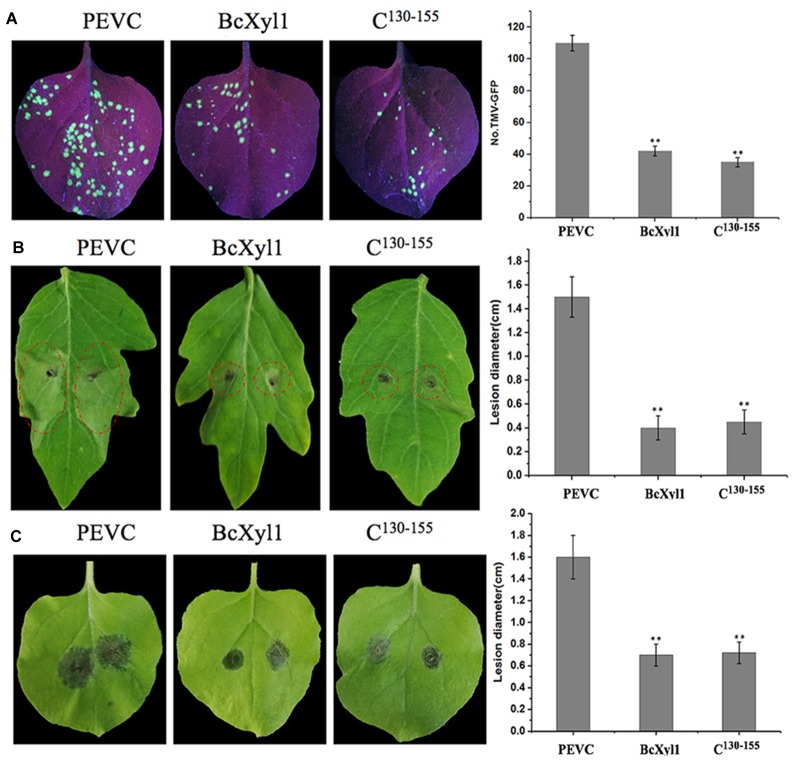
BcXyl1 confers disease resistance in plants. *N. benthamiana* or tomato leaves were infiltrated with 0.5 μM purified BcXyl1, C^130-155^, or PEVC. **(A)** The systemic leaves were inoculated with TMV-GFP, and the number of TMV-GFP lesions were measured. **(B,C)** The *N. benthamiana* or tomato systemic leaves were inoculated with 5 μL of 2 × 10^6^ conidia/ml *Botrytis cinerea*. Lesions symptoms and diameter were observed and measured at 2 days post-inoculation, respectively. Error bars represent standard deviation of three independent replicates. Student’s *t*-test was performed to determine the significant differences between BcXyl1, C^130-155^, and PEVC. Asterisks “^∗∗^” indicate statistically significant differences at a *p*-value <0.01.

Recent reports showed that fungi CWDEs could confer plants disease resistance (Gui Y. et al., 2017; Gui Y.-J. et al., 2017; Zhu et al., 2017). To further confirm the role of BcXyl1 in conferring resistance to plant diseases, the *N. benthamiana* leaves were treated with 1 μM BcXyl1or PEVC, and after 2 days, the systemic leaves were inoculated with TMV-GFP and *B. cinerea* spore suspension. BcXyl1-treated tobacco plants enhanced disease resistance against TMV, and the number of TMV-GFP lesions of BcXyl1-treated leaves was significantly decreased than that of the leaves treated with PEVC (Figure 6A). Meanwhile, BcXyl1 led to more resistance to the *B. cinerea* infection in *N. benthamiana*, as significantly lower lesions size on leaves compared with the leaves treated with PEVC controls (Figure 6B). Furthermore, in tomato plants that were pre-infiltrated with BcXyl1, lesion size on the *B. cinerea*-infected leaves was significant smaller compared with lesions size on leaves in plants that were pre-infiltrated with PEVC (Figure 6C). Together, these results strongly suggested that BcXyl1 conferred plants disease resistance.

### A Small Peptide of BcXyl1 Is Sufficient for Elicitor Function

**FIGURE 7:**
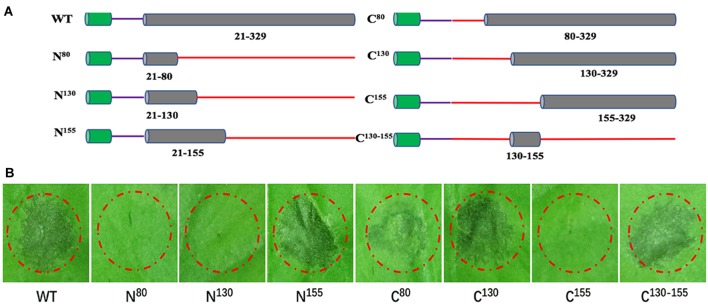
A small peptide of BcXyl1 is sufficient for inducing cell death. **(A)** Schematic presentation of the examined constructs, including N^80^, N^130^, N^155^, C^80^, C^130^, C^155^, and C^130-155^. **(B)** Analysis of cell death produced by *A. tumefaciens* strains transiently expressing various truncated mutants in *N. benthamiana* leaves. All the experiments were replicated three times.

The plant receptors often recognize specific small protein epitopes of PAMP to induce plant immunity (Rotblat et al., 2002). To delineate the elicitor active peptide of BcXyl1, we generated N-terminal and C-terminal truncated mutants and detected the ability to induce cell death by agroinfiltration in *N. benthamiana* leaves (Figure 7A). We found that the N-terminal truncated mutant (N^155^) maintained the ability of cell death-inducing, whereas expression of N^80^ and N^130^ did not trigger cell death. The C-terminal truncated mutants (C^80^ and C^130^) induced cell death, but C^155^ resulted in the loss of cell death-inducing activity in *N. benthamiana*. Further, C^130-155^ induced the same cell death symptom compared with full-length BcXyl1 in *N. benthamiana* (Figure 7B). Hence, C^130-155^ was identified as the functional peptide of BcXyl1 to induce cell death in *N. benthamiana*. To probe whether C^130-155^ induced plant immune responses, purified C^130-155^ was used to infiltrate plants leaves. We found that like BcXyl1, C^130-155^ could induce typical PTI responses, including accumulation of ROS, leakage of ion electrolytes, expression of defense genes, and callose deposition (Figure 5). Meanwhile, C^130-155^ could also enhance resistance to *B. cinerea* and TMV in plants (Figure 6). These results suggested that a small peptide of BcXyl1 was sufficient for elicitor function.

### BAK1 and SOBIR1 Mediates BcXyl1-Triggered Cell Death in *N. benthamiana*

**FIGURE 8:**
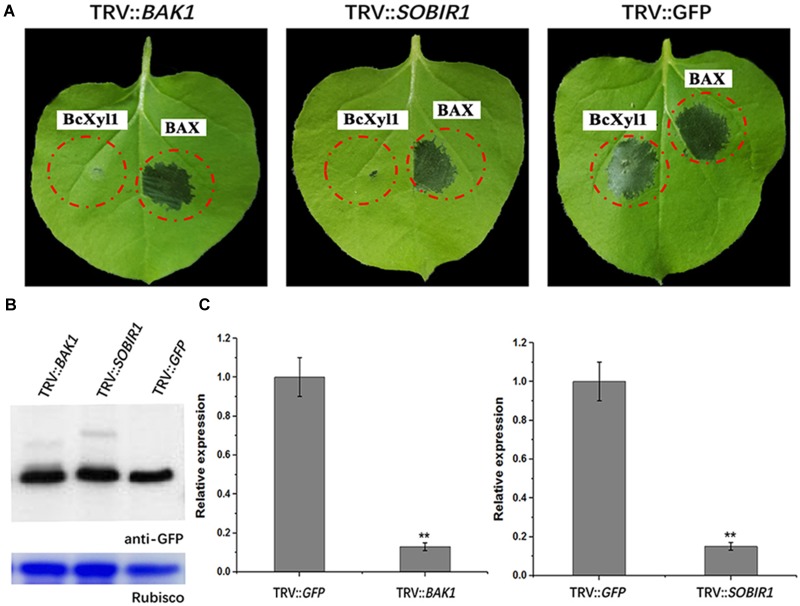
BAK1 and SOBIR1 mediates BcXyl1-triggered cell death in *N. benthamiana*. Three silence constructs (TRV::*BAK1*, TRV::*SOBIR1*, and TRV::*GFP*) were generated. **(A)** BcXyl1 and BAX (the positive control) were transiently expressed in *BAK1* and *SOBIR1* silenced tobacco leaves, respectively. The cell death induction was detected and photographed in *N. benthamiana* leaves 4 days after infiltration. **(B)** Immunoblot analysis of BcXyl1 transiently expressed in genes-silenced *N. benthamiana* leaves. **(C)** The silencing efficiency of *BAK1* and *SOBIR1* was examined by qPCR. Error bars represent standard errors. Error bars represent standard deviation of three independent replicates. Student’s *t*-test was performed to determine the significant differences between mutants and WT. Asterisks “^∗∗^” indicate statistically significant differences at a *p*-value <0.01.

The plant PRRs, such as the LRR RLKs BAK1 and SOBIR1, were employed to participate in multiple PRR pathways, including cell death induction (Monaghan and Zipfel, 2012; Liebrand et al., 2014; Gravino et al., 2016). For example, BAK1 was required for cell death inducing of GH12 members (Ma et al., 2015; Zhu et al., 2017). As demonstrated above, BcXyl1 was secreted into the apoplast to induce cell death. To determine whether BAK1 and SOBIR1 participated in induction of cell death by BcXyl1, we used virus-induced gene silencing (VIGS) to induce the gene silencing of *BAK1* or *SOBIR1* in *N*. *benthamiana* leaves. Three weeks after viral inoculation to silence *BAK1*, transient expression of BcXyl1 in *N. benthamiana* did not result in cell death after agroinfiltration with BcXyl1 expression constructs. Treatment of *BAK1*-silenced plants with Bcl-2-associated protein X (BAX) was used as a control, which resulted in cell death induction (Figure 8A). The results of *SOBIR1*-silenced plants were in accordance with BAK1-silenced plants, BcXyl1 did not trigger cell death, while BAX was still capable of inducing cell death (Figure 8A). Immunoblotting confirmed that BcXyl1 were successfully expressed at the expected size in *N. benthamiana* plants inoculated with TRV::*BAK*, TRV::*SOBIR1*, or TRV::*GFP* (Figure 8B). qPCR analysis confirmed that the expression of *BAK1* or *SOBIR1* expression was markedly reduced upon inoculation with the TRV::*BAK* or TRV::*SOBIR1*, with an expression level about 20% in comparison with inoculation with TRV::GFP (Figure 8C). From these results, we inferred that BAK1 and SOBIR1 (a LRR-RLP/SOBIR1/BAK1 complex) were required for BcXyl1-triggered cell death in *N. benthamiana*.

## Discussion

*Botrytis cinerea*, a necrotrophic plant pathogen, attacks the plant organs, including leaves, flowers, fruits, bulb, and root tubers, and causes serious plant diseases and substantial losses in agriculture throughout the world (Williamson et al., 2007; Ky et al., 2012). Like other phytopathogenic fungi, *B. cinerea* secretes vast array of proteins during infection process (Fillinger and Elad, 2016). Cell wall-degrading enzymes (CWDEs) are the largest class of *B. cinerea*-secreted proteins (Kubicek et al., 2014). Recent studies have revealed that several CWDEs functioned as virulence factors in plant pathogens and were also recognized as PAMPs by plant PRRs to trigger the PTI responses, during plant–pathogen interactions (Ma et al., 2015). In this study, we described the identification and analysis of BcXyl1, a secreted xylanase from *B. cinerea*. BcXyl1 had the ability to induce cell death and plant PTI responses independent of its enzymatic activity. Furthermore, our study also found that a small peptide from BcXyl1 was sufficient for elicitor activity. VIGS assays showed that a LRR-RLP/SOBIR1/BAK1 complex modulates BcXyl1-triggered cell death in *N. benthamiana*. We also found that BcXyl1 functions as a virulence factor that contributes to *B. cinerea* virulence on host plants.

Increasing evidence demonstrated that xylanases are responsible for the pathogenesis of necrotrophic phytopathogens, including *B. cinerea* (Schouten et al., 2007; Frías et al., 2011). For instance, xyn11A was an endo-β-1,4-xylanase belonging to family 11 of glycoside hydrolase and required for virulence in *B. cinerea*, and the deletion of the *xynB* gene encoding an endo-xylanase distinctly reduced the virulence of *Xanthomonas oryzae* pv. *oryzae* (Brito et al., 2006; Pandey and Sonti, 2010). In this study, we found BcXyl1 appeared to be a major virulence factor. Strikingly, *BcXyl1* was strongly induced and accumulated during the early stage of infection, and the mutation of *BcXyl1* had a severe effect on pathogenicity (Figures 1, 2). It is noteworthy that not all fungal xylanases have been conclusively involved in pathogenicity and virulence. So far, gene deletion experiments in *Fusarium oxysporum*, *Fusarium graminearum*, *Magnaporthe grisea*, and *Cochliobolus carbonum* did not support an essential role for xylanases in fungal pathogenesis (Apel, 1993; Wu et al., 1997; Gómez-Gómez et al., 2002; Santhanam et al., 2013; Sella et al., 2013). In addition, previously study showed that a xylanase from *B. cinerea* could contribute to virulence by promoting the necrosis of the plant tissue surrounding the infection (Noda et al., 2010). Interestingly, a few nanograms of purified BcXyl1 resulted in a rapid leaf tissue necrosis in soybean, tomato, cotton, and *N. benthamiana* (Figure 3C). The range of plant species responding to BcXyl1 may be larger than we detected.

Previous studies showed that the enzymatic activity of many fungal CWDEs was required for cell death-inducing activity (Gui Y. et al., 2017). However, in certain cases, the cell death inducing activity was found to be unrelated to the enzymatic activity (Ma et al., 2014, 2015). For instance, Xyn11A, a xylanase from *B. cinerea*, and the *Trichoderma viride* EIX could induce cell death in plants independent of the xylanase activity (Furman-Matarasso et al., 1999; Noda et al., 2010). Although BcXyl1 is a xylanase, induction of cell death did not require the enzymatic activity (Figure 4).

Our results showed that BcXyl1 was localized to the plant apoplast by a signal peptide experiment, suggesting that the cell death-inducing activity may be mediated by surface-localized PRRs (Supplementary Figure S5). Plant surface-localized PRRs such as RLKs and RLPs were involved in the recognition of PAMPs (Boutrot and Zipfel, 2017). In addition, BAK1, as a co-receptor, plays a regulatory role in receptor complexes that mediate PTI (Schulze et al., 2010; Liu et al., 2013; Gravino et al., 2016; Yamada et al., 2016). And SOBIR1 is also specifically required for the function of receptor complexes (Liebrand et al., 2014). VIGS assays confirmed that tobacco BAK1 was required for BcXyl1-induced cell death, and the cell-death response also disappeared in VIGS-*SOBIR1* plants (Figure 8). Hence, the RLP–SOBIR1–BAK1 complex mediated the cell death-inducing activity of BcXyl1.

The detection of PAMPs by plant PRRs to trigger PTI is a major component of plant defense responses. We confirmed that BcXyl1 triggered typical defense responses, including accumulation of ROS, leakage of ion electrolytes, deposition of callose, and expression of defense genes (Figure 5). We also found that the recombinant BcXyl1 proteins conferred systemic resistance in *N. benthamiana*, which offered protection against TMV and *B. cinerea* (Figure 6).

Generally, PAMPs are perceived by PRRs *via* specific epitopes, and the small peptides located on the surface of the proteins are sufficient to stimulate immune responses. For example, a 35-amino acid peptide of BcIEB1 could trigger necrosis and the PTI responses (González et al., 2017). Similarly, a 30-amino acid peptide of Xyn11A mediated the induction of cell death (Noda et al., 2010). The small peptide of VdEG3 from the GH12 domain was sufficient to induce cell death in *N. benthamiana* (Gui Y.-J. et al., 2017). In this study, progressive truncation of BcXyl1 confirmed that a region with 26 amino acids was sufficient for elicitor function (Figures 5–7).

Previous studies showed that many fungal xylanases involved in inducing plant defense responses immunity. For instance, the xylanase EIX from *T. viride* was an elicitor to induce defense responses in tomato, pepper and tomato plants (Rotblat et al., 2002). A xylanase from *F. graminearum* could induce cell death and hydrogen peroxide accumulation in wheat leaves (Sella et al., 2013; Moscetti et al., 2014). We have also determined that BcXyl1 induced plant defense responses and conferred tobacco and tomato plants disease resistance. Therefore, we speculated that fungal xylanases have the ability to trigger immunity in dicot and monocot plants.

Successful pathogens deliver effectors to interference the host PTI response and establish infection (Jones and Dangl, 2006; Gimenez-Ibanez et al., 2009). For example, a RXLR effector and CBM1 effector suppressed XEG1-triggered immunity in oomycetes and suppressed the GH12 protein and BcXyl1-triggered immunity in *V. dahlia*, respectively. Whether effectors mediate the suppression of BcXyl1, needs further investigation.

## Author Contributions

YD and DQ designed the experiments. YY performed most of the experiments and wrote the paper. XY participated in some part of the study and the Graduate Student Innovation Scientific Research Subject of Hainan Province (Hyb2017-17).

## Conflict of Interest Statement

The authors declare that the research was conducted in the absence of any commercial or financial relationships that could be construed as a potential conflict of interest.

## Supplementary Material

FIGURE S1 Sequence alignment of BcXyl1 and xylanases from other fungi. Two red triangles indicated possible catalytic residues of BcXyl1 (E104 and E157). Sequence data of all proteins can be found in the GenBank/EMBL data libraries under accession numbers: XynBc1 (ACF16413.1), BcXyl1 (ATZ53308.1), XynG1 (XP_001258363.1), BcXyl2 (XP_001546507.1), BcXyl3 (ATZ58346.1), BcXyl4 (ATZ51455.1), NpGH11 (EOD46026.1), and SsGH11 (XP_001588545).

FIGURE S2 *BcXyl1* deletion strains do not show developmental defects. *BcXyl1* deletion mutants (Δ*BcXyl1*-1 and Δ*BcXyl1*-2), rescued strains (Δ*BcXyl1*-1-C and Δ*BcXyl1*-2-C). **(A)** The radial growth and colony morphology were observed after 8 days of incubation on PDA medium at 25°C. **(B)** Conidial germination rate of each strain was determined after cultivated on Water-Agar media at 25°C for 15 h. **(C)** Fungi were grown on PDA plates at 25°C. Radial growth was measured every day, and the growth rate was calculated. All the experiments were replicated three times.

FIGURE S3 SDS-PAGE analysis of BcXyl1 and BcXyl1^rec^ recombinant proteins. BcXyl1 is the native protein; BcXyl1^rec^ is the site-directed mutagenized protein, which E104 and E157 were substituted with Gln. Two recombinant proteins were stained with Coomassie blue.

FIGURE S4 BcXyl1 is secreted into the apoplast to induce cell death. **(A)** BcXyl1 (the native protein) and BcXyl1^-ΔSP^ (deleted the signal peptide). Cell death induction was detected in *N. benthamiana* leaves 5 days after infiltration with the examined various *A. tumefaciens* strains. **(B)** Immunoblot analysis of proteins from *N. benthamiana* leaves transiently expressing the examined proteins using a pYBA1132 vector. All the experiments were replicated three times.

FIGURE S5 BcXyl1 confers disease resistance in plants. *N. benthamiana* or tomato leaves were infiltrated with 0.5 μM purified BcXyl1, C^130-155^, or PEVC. **(A)** The local leaves were inoculated with TMV-GFP, and the number of TMV-GFP lesions were measured. **(B,C)** The *N. benthamiana* or tomato local leaves were inoculated with 5 μL of 2 × 10^6^ conidia/ml *Botrytis cinerea*. Lesions symptoms and diameter were observed and measured at 2 days post-inoculation, respectively. Error bars represent standard deviation of three independent replicates. Student’s *t*-test was performed to determine the significant differences between BcXyl1, C^130-155^ and PEVC. Asterisks “^∗∗^” indicate statistically significant differences at a *p*-value <0.01.

TABLE S1 Hydrolysis activity test.

TABLE S2 Primers used in this study.

## References

[B1] Apel P. C. (1993). Cloning and targeted gene disruption of XYL1,a β1,4-Xylanase gene from the maize pathogen *Cochliobolus carbonum*. *Mol. Plant Microbe Interact.* 6 467–473. 10.1094/MPMI-6-467 8400376

[B2] Ben-Daniel B.-H. Bar-Zvi D. Tsror Lahkim L. (2011). Pectate lyase affects pathogenicity in natural isolates of *Colletotrichum coccodes* and in pelA gene-disrupted and gene-overexpressing mutant lines. *Mol. Plant Pathol.* 13 187–197. 10.1111/j.1364-3703.2011.00740.x 21848609 PMC6638648

[B3] Benito E. P. ten Have A. van ’t Klooster J. W. van Kan J. A. L. (1998). Fungal and plant gene expression during synchronized infection of tomato leaves by *Botrytis cinerea*. *Eur. J. Plant Pathol.* 104 207–220. 10.1023/A:1008698116106

[B4] Biely P. Mislovičová D. Toman R. (1988). Remazol brilliant blue-xylan: a soluble chromogenic substrate for xylanases. *Meth. Enzymol.* 160 536–541. 10.1016/0076-6879(88)60165-0

[B5] Bindschedler L. V. Dewdney J. Blee K. A. Stone J. M. Asai T. Plotnikov J. (2006). Peroxidase-dependent apoplastic oxidative burst in *Arabidopsis* required for pathogen resistance. *Plant J.* 47 851–863. 10.1111/j.1365-313X.2006.02837.x 16889645 PMC3233234

[B6] Boller T. Felix G. (2009). A renaissance of elicitors: perception of microbe-associated molecular patterns and danger signals by pattern-recognition receptors. *Annu. Rev. Plant Biol.* 60 379–406. 10.1146/annurev.arplant.57.032905.105346 19400727

[B7] Boutrot F. Zipfel C. (2017). Function, discovery, and exploitation of plant pattern recognition receptors for broad-spectrum disease resistance. *Annu. Rev. Phytopathol.* 55 257–286. 10.1146/annurev-phyto-080614-120106 28617654

[B8] Brito N. Espino J. J. González C. (2006). The endo-beta-1,4-xylanase xyn11A is required for virulence in *Botrytis cinerea*. *MPMI* 19 25–32. 10.1094/MPMI-19-0025 16404950

[B9] Brutus A. Reca I. B. Herga S. Mattei B. Puigserver A. Chaix J.-C. (2005). A family 11 xylanase from the pathogen *Botrytis cinerea* is inhibited by plant endoxylanase inhibitors XIP-I and TAXI-I. *Biochem. Biophys. Res. Commun.* 337 160–166. 10.1016/j.bbrc.2005.09.030 16185656

[B10] Cantarel B. L. Coutinho P. M. Rancurel C. Bernard T. Lombard V. Henrissat B. (2009). The carbohydrate-active enzymes database (CAZy): an expert resource for glycogenomics. *Nucleic Acids Res.* 37 D233–D238. 10.1093/nar/gkn663 18838391 PMC2686590

[B11] Chisholm S. T. Coaker G. Day B. Staskawicz B. J. (2006). Host-microbe interactions: shaping the evolution of the plant immune response. *Cell* 124 803–814. 10.1016/j.cell.2006.02.008 16497589

[B12] Chen M. Zeng H. Qiu D. Guo L. Yang X. Shi H. (2012). Purification and characterization of a novel hypersensitive response-inducing elicitor from *Magnaporthe oryzae* that triggers defense response in rice. *PLoS One* 7:e37654. 10.1371/journal.pone.0037654 22624059 PMC3356297

[B13] Collins T. Gerday C. Feller G. (2005). Xylanases, xylanase families and extremophilic xylanases. *FEMS Microbiol. Rev.* 29 3–23. 10.1016/j.femsre.2004.06.005 15652973

[B14] Couto D. Zipfel C. (2016). Regulation of pattern recognition receptor signalling in plants. *Nat. Rev. Immunol.* 16 537–552. 10.1038/nri.2016.77 27477127

[B15] Fillinger S. Elad Y. (2016). *Botrytis – the Fungus, the Pathogen and its Management in Agricultural Systems*. Berlin: Springer International Publishing 10.1007/978-3-319-23371-0

[B16] Frías M. Brito N. González C. (2012). The *Botrytis cinerea* cerato-platanin BcSpl1 is a potent inducer of systemic acquired resistance (SAR) in tobacco and generates a wave of salicylic acid expanding from the site of application. *Mol. Plant Pathol.* 14 191–196. 10.1111/j.1364-3703.2012.00842.x 23072280 PMC6638659

[B17] Frías M. González C. Brito N. (2011). BcSpl1, a cerato-platanin family protein, contributes to *Botrytis cinerea* virulence and elicits the hypersensitive response in the host. *New Phytol.* 192 483–495. 10.1111/j.1469-8137.2011.03802.x 21707620

[B18] Furman-Matarasso N. Cohen E. Du Q. Chejanovsky N. Hanania U. Avni A. (1999). A point mutation in the ethylene-inducing xylanase elicitor inhibits the beta-1-4-endoxylanase activity but not the elicitation activity. *Plant Physiol.* 121 345–351. 10.1104/pp.121.2.345 10517825 PMC59396

[B19] Gimenez-Ibanez S. Hann D. R. Ntoukakis V. Petutschnig E. Lipka V. Rathjen J. P. (2009). AvrPtoB targets the LysM receptor kinase CERK1 to promote bacterial virulence on plants. *Curr. Biol.* 19 423–429. 10.1016/j.cub.2009.01.054 19249211

[B20] Gómez-Gómez E. Ruíz-Roldán M. C. Di Pietro A. Roncero M. I. G. Hera C. (2002). Role in pathogenesis of two endo-β-1,4-xylanase genes from the vascular wilt fungus *Fusarium oxysporum*. *Fungal Genet. Biol.* 35 213–222. 10.1006/fgbi.2001.1318 11929211

[B21] González M. Brito N. González C. (2017). The *Botrytis cinerea* elicitor protein BcIEB1 interacts with the tobacco PR5-family protein osmotin and protects the fungus against its antifungal activity. *New Phytol.* 215 397–410. 10.1111/nph.14588 28480965

[B22] Gravino M. Locci F. Tundo S. Cervone F. Savatin D. V. De Lorenzo G. (2016). Immune responses induced by oligogalacturonides are differentially affected by AvrPto and loss of BAK1/BKK1 and PEPR1/PEPR2. *Mol. Plant Pathol.* 18 582–595. 10.1111/mpp.12419 27118426 PMC6638274

[B23] Gui Y. Zhang W. Zhang D. Zhou L. Short D. P. G. Wang J. (2017). A *Verticillium dahliae* extracellular cutinase modulates plant immune responses. *Mol. Plant Microbe Interact.* 31 260–273. 10.1094/MPMI-06-17-0136-R 29068240

[B24] Gui Y.-J. Chen J.-Y. Zhang D.-D. Li N.-Y. Li T.-G. Zhang W.-Q. (2017). *Verticillium dahliae* manipulates plant immunity by glycoside hydrolase 12 proteins in conjunction with carbohydrate-binding module 1. *Environ. Microbiol.* 19 1914–1932. 10.1111/1462-2920.13695 28205292

[B25] Houterman P. M. Cornelissen B. J. C. Rep M. (2008). Suppression of plant resistance gene-based immunity by a fungal effector. *PLoS Pathog.* 4:e1000061. 10.1371/journal.ppat.1000061 18464895 PMC2330162

[B26] Jones J. D. G. Dangl J. L. (2006). The plant immune system. *Nature* 444 323–329. 10.1038/nature05286 17108957

[B27] Kettles G. J. Bayon C. Canning G. Rudd J. J. Kanyuka K. (2016). Apoplastic recognition of multiple candidate effectors from the wheat pathogen *Zymoseptoria triticiin* the nonhost plant *Nicotiana benthamiana*. *New Phytol.* 213 338–350. 10.1111/nph.14215 27696417 PMC5132004

[B28] Kubicek C. P. Starr T. L. Glass N. L. (2014). Plant cell wall–degrading enzymes and their secretion in plant-pathogenic fungi. *Annu. Rev. Phytopathol.* 52 427–451. 10.1146/annurev-phyto-102313-045831 25001456

[B29] Ky I. Lorrain B. Jourdes M. Pasquier G. Fermaud M. Gény L. (2012). Assessment of grey mould (*Botrytis cinerea*) impact on phenolic and sensory quality of *Bordeaux grapes*, musts and wines for two consecutive vintages. *Aust. J. Grape Wine Res.* 18 215–226. 10.1111/j.1755-0238.2012.00191.x

[B30] Liebrand T. W. H. van den Burg H. A. Joosten M. H. A. J. (2014). Two for all: receptor-associated kinases SOBIR1 and BAK1. *Trends Plant Sci.* 19 123–132. 10.1016/j.tplants.2013.10.003 24238702

[B31] Liu Z. Wu Y. Yang F. Zhang Y. Chen S. Xie Q. (2013). BIK1 interacts with PEPRs to mediate ethylene-induced immunity. *Proc. Natl. Acad. Sci. U.S.A.* 110 6205–6210. 10.1073/pnas.1215543110 23431184 PMC3625333

[B32] Livak K. J. Schmittgen T. D. (2001). Analysis of relative gene expression data using real-time quantitative PCR and the 2–ΔΔCT method. *Methods* 25 402–408. 10.1006/meth.2001.1262 11846609

[B33] Ma Y. Han C. Chen J. Li H. He K. Liu A. (2014). Fungal cellulase is an elicitor but its enzymatic activity is not required for its elicitor activity. *Mol. Plant Pathol.* 16 14–26. 10.1111/mpp.12156 24844544 PMC6638370

[B34] Ma Z. Song T. Zhu L. Ye W. Wang Y. Shao Y. (2015). A *Phytophthora sojae* glycoside hydrolase 12 protein is a major virulence factor during soybean infection and is recognized as a PAMP. *Plant Cell* 27 2057–2072. 10.1105/tpc.15.00390 26163574 PMC4531360

[B35] Monaghan J. Zipfel C. (2012). Plant pattern recognition receptor complexes at the plasma membrane. *Curr. Opin. Plant Biol.* 15 349–357. 10.1016/j.pbi.2012.05.006 22705024

[B36] Moscetti I. Faoro F. Moro S. Sabbadin D. Sella L. Favaron F. (2014). The xylanase inhibitor TAXI-III counteracts the necrotic activity of a *Fusarium graminearum* xylanase in vitroand in durum wheat transgenic plants. *Mol. Plant Pathol.* 16 583–592. 10.1111/mpp.12215 25346411 PMC6638430

[B37] Noda J. Brito N. González C. (2010). The *Botrytis cinerea* xylanase Xyn11A contributes to virulence with its necrotizing activity, not with its catalytic activity. *BMC Plant Biol.* 10:38. 10.1186/1471-2229-10-38 20184750 PMC2844071

[B38] Pandey A. Sonti R. V. (2010). Role of the FeoB protein and siderophore in promoting virulence of *Xanthomonas oryzae* pv. *oryzae on Rice*. *J. Bacteriol.* 192 3187–3203. 10.1128/JB.01558-09 20382771 PMC2901680

[B39] Prins T. W. Tudzynski P. von Tiedemann A. Tudzynski B. ten Have A. Hansen M. E. (2000). “Infection strategies of *Botrytis cinerea* and related necrotrophic pathogens,” in *Fungal Pathology*, ed. Kronstad J. W. (Dordrecht: Kluwer Academic Publishers Group), 33–64.

[B40] Rotblat B. Enshell-Seijffers D. Gershoni J. M. Schuster S. Avni A. (2002). Identification of an essential component of the elicitation active site of the EIX protein elicitor. *Plant J.* 32 1049–1055. 10.1046/j.1365-313X.2002.01490.x 12492845

[B41] Saijo Y. Loo E. P.-I. Yasuda S. (2017). Pattern recognition receptors and signaling in plant-microbe interactions. *Plant J.* 93 592–613. 10.1111/tpj.13808 29266555

[B42] Santhanam P. van Esse H. P. Albert I. Faino L. Nürnberger T. Thomma B. P. H. J. (2013). Evidence for functional diversification within a fungal NEP1-Like protein family. *MPMI* 26 278–286. 10.1094/MPMI-09-12-0222-R 23051172

[B43] Schouten A. van Baarlen P. van Kan J. A. L. (2007). Phytotoxic Nep1-like proteins from the necrotrophic fungus *Botrytis cinerea* associate with membranes and the nucleus of plant cells. *New Phytol.* 177 493–505. 18028294 10.1111/j.1469-8137.2007.02274.x

[B44] Schulze B. Mentzel T. Jehle A. K. Mueller K. Beeler S. Boller T. (2010). Rapid heteromerization and phosphorylation of ligand-activated plant transmembrane receptors and their associated kinase BAK1. *J. Biol. Chem.* 285 9444–9451. 10.1074/jbc.M109.096842 20103591 PMC2843194

[B45] Sella L. Gazzetti K. Faoro F. Odorizzi S. D’Ovidio R. Schäfer W. (2013). A *Fusarium graminearum* xylanase expressed during wheat infection is a necrotizing factor but is not essential for virulence. *Plant Physiol. Biochem.* 64 1–10. 10.1016/j.plaphy.2012.12.008 23337356

[B46] Stergiopoulos I. de Wit P. J. G. M. (2009). Fungal effector proteins. *Annu. Rev. Phytopathol.* 47 233–263. 10.1146/annurev.phyto.112408.132637 19400631

[B47] Williamson B. Tudzynski B. Tudzynski P. van Kan J. A. L. (2007). *Botrytis cinerea*: the cause of grey mould disease. *Mol. Plant Pathol.* 8 561–580. 10.1111/j.1364-3703.2007.00417.x 20507522

[B48] Wu S.-C. Ham K.-S. Darvill A. G. Albersheim P. (1997). Deletion of two endo-β-1,4-Xylanase genes reveals additional isozymes secreted by the rice blast fungus. *MPMI* 10 700–708. 10.1094/MPMI.1997.10.6.700

[B49] Yakoby N. Beno-Moualem D. Keen N. T. Dinoor A. Pines O. Prusky D. (2001). *Colletotrichum gloeosporioides* pelBIs an important virulence factor in avocado fruit-fungus interaction. *MPMI* 14 988–995. 10.1094/MPMI.2001.14.8.988 11497471

[B50] Yamada K. Yamashita-Yamada M. Hirase T. Fujiwara T. Tsuda K. Hiruma K. (2016). Danger peptide receptor signaling in plants ensures basal immunity upon pathogen-induced depletion of BAK1. *EMBO J.* 35 46–61. 10.15252/embj.201591807 26574534 PMC4718002

[B51] Yu X. Tang J. Wang Q. Ye W. Tao K. Duan S. (2012). The RxLR effector Avh241 from *Phytophthora sojae* requires plasma membrane localization to induce plant cell death. *New Phytol.* 196 247–260. 10.1111/j.1469-8137.2012.04241.x 22816601

[B52] Zhang H. Wu Q. Cao S. Zhao T. Chen L. Zhuang P. (2014). A novel protein elicitor (SsCut) from *Sclerotinia sclerotiorum* induces multiple defense responses in plants. *Plant Mol. Biol.* 86 495–511. 10.1007/s11103-014-0244-3 25149470

[B53] Zhang Y. Zhang Y. Qiu D. Zeng H. Guo L. Yang X. (2015). BcGs1, a glycoprotein from *Botrytis cinerea*, elicits defence response and improves disease resistance in host plants. *Biochem. Biophys. Res. Commun.* 457 627–634. 10.1016/j.bbrc.2015.01.038 25613865

[B54] Zhu W. Ronen M. Gur Y. Minz-Dub A. Masrati G. Ben-Tal N. (2017). BcXYG1, a secreted xyloglucanase from *Botrytis cinerea*, triggers both cell death and plant immune responses. *Plant Physiol.* 175 438–456. 10.1104/pp.17.00375 28710128 PMC5580746

[B55] Zipfel C. (2008). Pattern-recognition receptors in plant innate immunity. *Curr. Opin. Immunol.* 20 10–16. 10.1016/j.coi.2007.11.003 18206360

