# Supplementary figures and images for: The Botrytis cinerea Xylanase BcXyl1 Modulates Plant Immunity
Source: Front Microbiol. 2018 Oct 23;9:2535. doi: 10.3389/fmicb.2018.02535 (PMC6206051; doi:10.3389/fmicb.2018.02535)

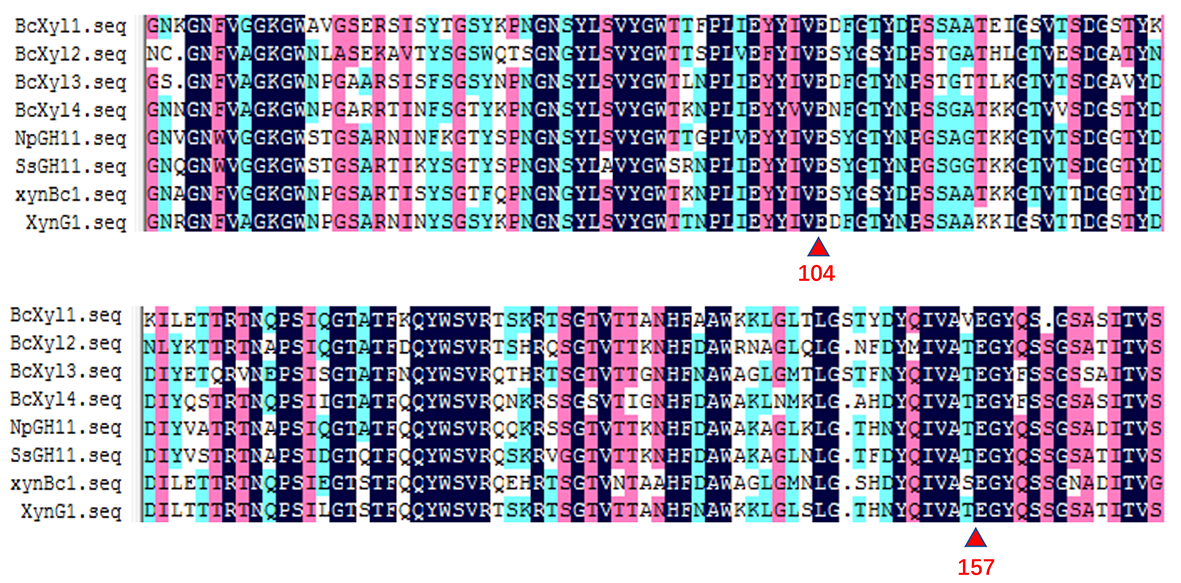

Supplement: FIGURE S1 — Sequence alignment of BcXyl1 and xylanases from other fungi. Two red triangles indicated possible catalytic residues of BcXyl1 (E104 and E157). Sequence data of all proteins can be found in the GenBank/EMBL data libraries under accession numbers: XynBc1 (ACF16413.1), BcXyl1 (ATZ53308.1), XynG1 (XP_001258363.1), BcXyl2 (XP_001546507.1), BcXyl3 (ATZ58346.1), BcXyl4 (ATZ51455.1), NpGH11 (EOD46026.1), and SsGH11 (XP_001588545). [file Image_1.TIF]

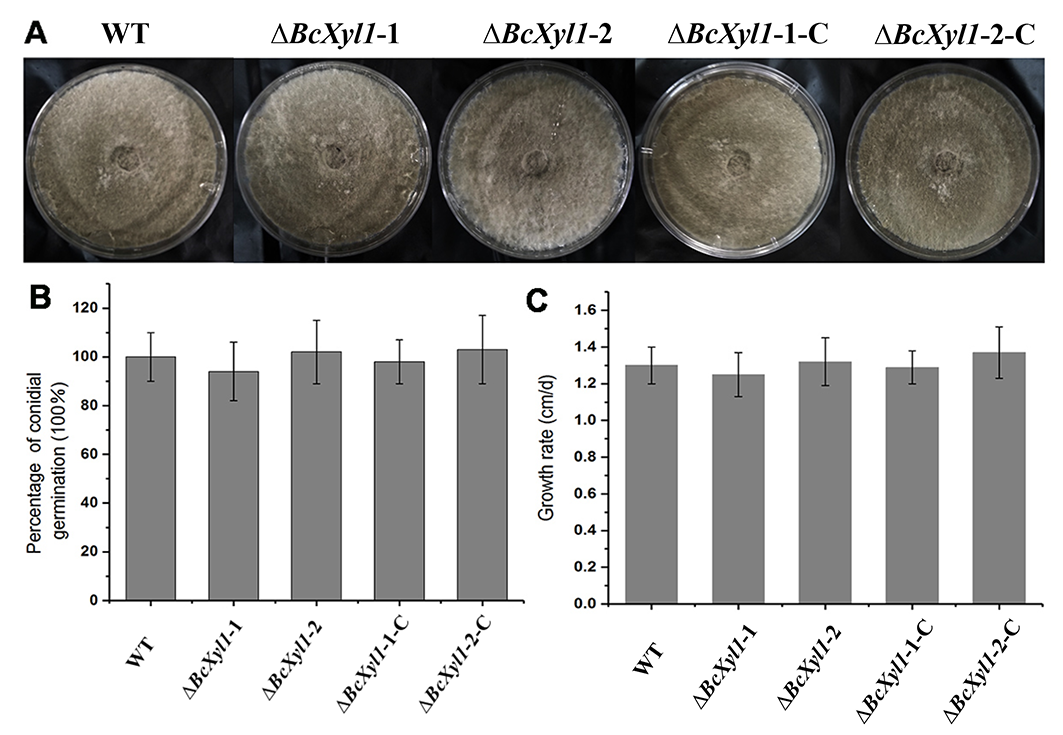

Supplement: FIGURE S2 — BcXyl1 deletion strains do not show developmental defects. BcXyl1 deletion mutants (ΔBcXyl1-1 and ΔBcXyl1-2), rescued strains (ΔBcXyl1-1-C and ΔBcXyl1-2-C). (A) The radial growth and colony morphology were observed after 8 days of incubation on PDA medium at 25°C. (B) Conidial germination rate of each strain was determined after cultivated on Water-Agar media at 25°C for 15 h. (C) Fungi were grown on PDA plates at 25°C. Radial growth was measured every day, and the growth rate was calculated. All the experiments were replicated three times. [file Image_2.TIF]

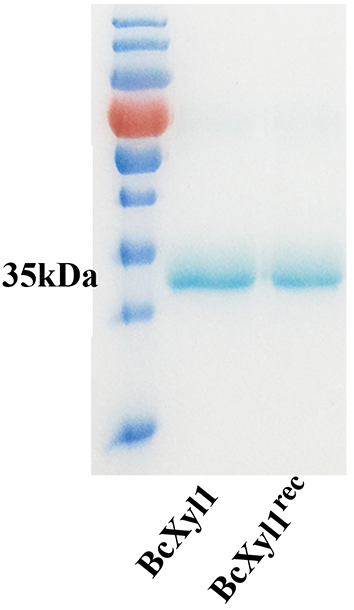

Supplement: FIGURE S3 — SDS-PAGE analysis of BcXyl1 and BcXyl1rec recombinant proteins. BcXyl1 is the native protein; BcXyl1rec is the site-directed mutagenized protein, which E104 and E157 were substituted with Gln. Two recombinant proteins were stained with Coomassie blue. [file Image_3.TIF]

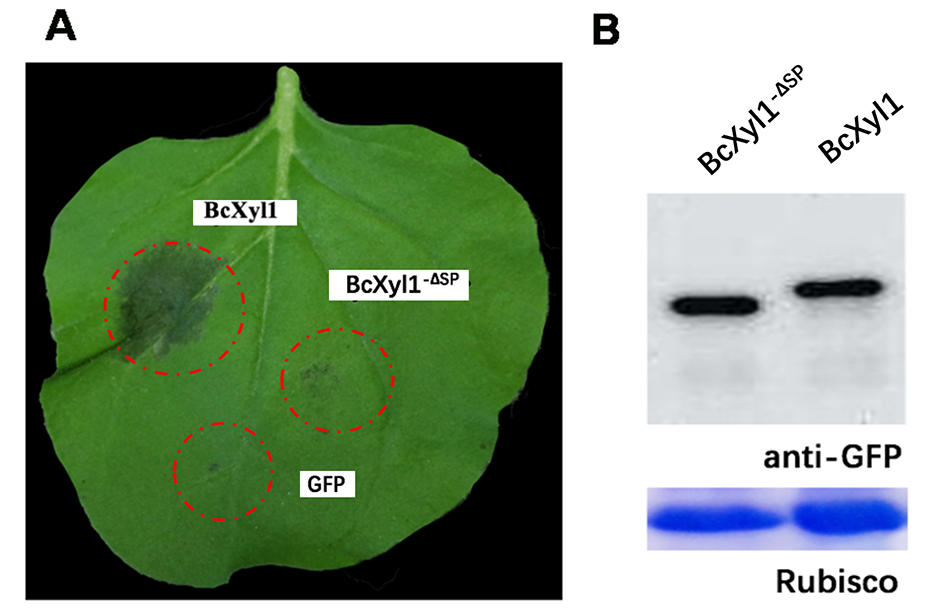

Supplement: FIGURE S4 — BcXyl1 is secreted into the apoplast to induce cell death. (A) BcXyl1 (the native protein) and BcXyl1-ΔSP (deleted the signal peptide). Cell death induction was detected in N. benthamiana leaves 5 days after infiltration with the examined various A. tumefaciens strains. (B) Immunoblot analysis of proteins from N. benthamiana leaves transiently expressing the examined proteins using a pYBA1132 vector. All the experiments were replicated three times. [file Image_4.TIF]

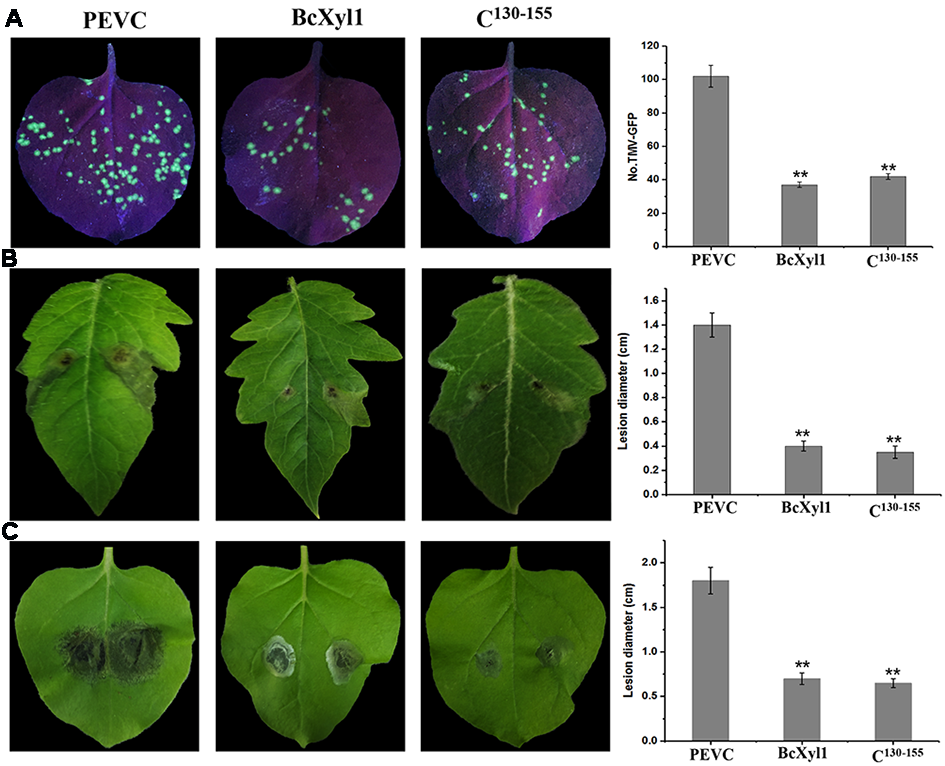

Supplement: FIGURE S5 — BcXyl1 confers disease resistance in plants. N. benthamiana or tomato leaves were infiltrated with 0.5 μM purified BcXyl1, C130-155, or PEVC. (A) The local leaves were inoculated with TMV-GFP, and the number of TMV-GFP lesions were measured. (B,C) The N. benthamiana or tomato local leaves were inoculated with 5 μL of 2 × 10^6^ conidia/ml Botrytis cinerea. Lesions symptoms and diameter were observed and measured at 2 days post-inoculation, respectively. Error bars represent standard deviation of three independent replicates. Student’s t-test was performed to determine the significant differences between BcXyl1, C130-155 and PEVC. Asterisks “∗∗” indicate statistically significant differences at a p-value <0.01. [file Image_5.TIF]
