# Supplementary material for: The Botrytis cinerea Xylanase BcXyl1 Modulates Plant Immunity
Source: Front Microbiol. 2018 Oct 23;9:2535. doi: 10.3389/fmicb.2018.02535 (PMC6206051; doi:10.3389/fmicb.2018.02535)
Supplement: TABLE S1 — Hydrolysis activity test. [file Table_1.pdf]

Table S1 Hydrolysis activity of BcXyl1 and BcXyl1^{rec}

Protein	Units/mg	(%)
BcXyl1	9.97±0.7	100±6.6
BcXyl1^{rec}	0.54±0.05	2.3±0.54

Values represent the averages of three independent measurements with three replicates each. Standard errors are shown.
