# Supplementary material for: The Botrytis cinerea Xylanase BcXyl1 Modulates Plant Immunity
Source: Front Microbiol. 2018 Oct 23;9:2535. doi: 10.3389/fmicb.2018.02535 (PMC6206051; doi:10.3389/fmicb.2018.02535)
Supplement: TABLE S2 — Primers used in this study. [file Table_2.pdf]

Table S2 Information of primers used in this study

Primer name	Primer 5'-3	Purpose
BcXyl1 F	CGGGATCCGATATTTCCC AATGTAAATC C	To amplify the DNA fragment of BcXyl1 for expression in *Pichia pastoris*
BcXyl1 R	CGGAATTC GCATAAAAGT AATTATCCGA GGTAGT	To amplify the DNA fragment of BcXyl1 for expression in *Pichia pastoris*
BcXyl1rec F	TATTGTGCGCGGTGGAGGAGCCCAGTCTGCTTCTGCAAAGGTTATCCAGCA	To amplify the DNA fragment of site-directed mutagenized BcXyl1rec for expression in *Pichia pastoris*
BcXyl1rec R	CGCGCACAATAGCGTTACCATCCCCCT TAATACTCAG CGCTGCCTCGCAGACA	To amplify the DNA fragment of site-directed mutagenized BcXyl1rec for expression in *Pichia pastoris*
BcXyl1 21-329-F	CGGAATCCGTCAACAACGTCACCCCCAACA	To transient expression BcXyl1 21-329 protein (deleted the N-terminal signal peptide)
BcXyl1 21-329-R	GCTCTAGA GCATAAAAGT AATTATCCGA GGTAGT	To transient expression BcXyl1 21-329 protein (deleted the N-terminal signal peptide)
BcXyl1-T-F	CGGAATCCATGCATTCCCTTCTTTATGCTAA CA	To transient expression BcXyl1 protein (with the N-terminal signal peptide)
BcXyl1-T-R	GCTCTAGA GCATAAAAGT AATTATCCGA GGTAGT	To transient expression BcXyl1 protein (with the N-terminal signal peptide)
RT-qPCR-NbEF1α-F	AGGATACAACCCTGACAAGA	*N. benthamiana EF-1α* gene used as qRT-PCR reference
RT-qPCR-NbEF1α-R	AGGATACAACCCTGACAAGA	*N. benthamiana EF-1α* gene used as qRT-PCR reference
RT-qPCR-Bcgpdh-F	CGAGTACCAGGCTGGTATCT	*B. cinerea Bcgpdh* gene used for qPCR measurement of pathogen levels and as qRT-PCR reference
RT-qPCR-Bcgpdh-R	TCATTGGTGGTTAGCCATCTT	*B. cinerea Bcgpdh* gene used for qPCR measurement of pathogen levels and as qRT-PCR reference
RT-qPCR-PR1a-F	GTGGGTCGATGAGAAACAGTAT	RT-qPCR for validation of *PR1a* gene transcription level in *N. benthamiana* leaves
RT-qPCR-PR1a-R	GAACCCTAGCACATCCAACA	RT-qPCR for validation of *PR1a* gene transcription level in *N. benthamiana* leaves
RT-qPCR-NPR1-F	GGAGCAAGCAGAAAGAAGAGA	RT-qPCR for validation of *NPR1* gene transcription level in *N. benthamiana* leaves
RT-qPCR-NPR1-R	GTTTAGCCAGGCCAACTCTAT	RT-qPCR for validation of *NPR1* gene transcription level in *N. benthamiana* leaves
RT-qPCR-PR5-F	GGGCCAATCTTGGAGCATTA	RT-qPCR for validation of *PR5* gene transcription level in *N. benthamiana* leaves
RT-qPCR-PR5-R	CAGTCTCCAGTCTCACAATTACC	RT-qPCR for validation of *PR5* gene transcription level in *N. benthamiana* leaves
RT-qPCR-COI1-F	GGCTTGACGTACTTAGGGAAATA	RT-qPCR for validation of *COI1* gene transcription level in *N. benthamiana* leaves
RT-qPCR-COI1-R	GGGACACCTTTGCAGTAAGA	RT-qPCR for validation of *COI1* gene transcription level in *N. benthamiana* leaves
RT-qPCR-PAL-F	ATTGCTGGTTTGCTCACTGG	RT-qPCR for validation of *PAL* gene transcription level in *N. benthamiana* leaves
RT-qPCR-PAL-R	TCCTTAGGCTGCAACTCGAA	RT-qPCR for validation of *PAL* gene transcription level in *N. benthamiana* leaves
NbBAK1-F	GGAATCCGTGAGGGTGGTGAGCGGGATAAT	Silencing of *BAK1* in *N. benthamiana*
NbBAK1-R	CCTCAGAGCTCATAACTGGGCAAAGGGCTT	Silencing of *BAK1* in *N. benthamiana*
NbSOBIR1-F	TGCTCTAGAGGAAGAAAAGGAAAGTCAGA	Silencing of Nb*SOBIR1* in *N. benthamiana*
NbSOBIR1-R	CCGCTCGAGATCCATTGGGGGTTGTATAA	Silencing of Nb*SOBIR1* in *N. benthamiana*
